# A novel method for identifying and distinguishing *Cryptococcus neoformans* and *Cryptococcus gattii* by surface-enhanced Raman scattering using positively charged silver nanoparticles

**DOI:** 10.1038/s41598-020-68978-0

**Published:** 2020-07-27

**Authors:** Shan Hu, Feng Gu, Min Chen, Chongwen Wang, Jia Li, Jian Yang, Guangyu Wang, Zhe Zhou, Ying Yang

**Affiliations:** 1 Department of Biotechnology, Beijing Institute of Radiation Medicine, Beijing Key Laboratory of New Molecular Diagnosis Technologies for Infectious Diseases, Beijing, 100850 People’s Republic of China; 2 Department of Laboratory Medicine, Xuzhou Tumor Hospital, Xuzhou, 221005 People’s Republic of China; 3 Shanghai Key Laboratory of Molecular Medical Mycology, Department of Dermatology, Changzheng Hospital, Second Military Medical University, Shanghai, People’s Republic of China; 4 College of Life Sciences, Anhui Agricultural University, Hefei, 230036 People’s Republic of China; 5 Department of Laboratory Medicine, Nanjing Drum Tower Hospital, The Affiliated Hospital of Nanjing University Medical School, Nanjing, 210008 People’s Republic of China; 6 Division of Recombinant Biological Products, National Institutes for Food and Drug Control, Beijing, 102629 People’s Republic of China

**Keywords:** Computational biology and bioinformatics, Microbiology, Diseases, Chemistry

## Abstract

There are approximately 1 million cryptococcal infections per year among HIV+ individuals, resulting in nearly 625,000 deaths. *Cryptococcus neoformans* and *Cryptococcus gattii* are the two most common species that cause human cryptococcosis. These two species of *Cryptococcus* have differences in pathogenicity, diagnosis, and treatment. Cryptococcal infections are usually difficult to identify because of their slow growth in vitro. In addition, the long detection cycle of *Cryptococcus* in clinical specimens makes the diagnosis of Cryptococcal infections difficult. Here, we used positively charged silver nanoparticles (AgNPs^+^) as a substrate to distinguish between *C. neoformans* and *C. gattii* in clinical specimens directly via surface-enhanced Raman scattering (SERS) and spectral analysis. The AgNPs^+^ self-assembled on the surface of the fungal cell wall via electrostatic aggregation, leading to enhanced SERS signals that were better than the standard substrate negatively charged silver nanoparticles (AgNPs). The SERS spectra could also be used as a sample database in the multivariate analysis via orthogonal partial least-squares discriminant analysis. This novel SERS detection method can clearly distinguish between the two Cryptococcus species using principal component analysis. The accuracy of the training data and test data was 100% after a tenfold crossover validation.

## Introduction

A report by the United Nation's HIV/AIDS (Human Immunodeficiency Virus/Acquired Immunodeficiency Syndrome) program and the World Health Organization (https://www.unaids.org/) in December 2018 showed that 37.9 million people were living with HIV/AIDS; 1.7 million individuals were newly infected with the disease in 2018, and 770,000 died of AIDS-related causes. On average, there are 1 million cryptococcal infections per year among individuals living with HIV/AIDS, resulting in nearly 625,000 deaths (source: Center for Disease Control, Atlanta, USA, https://www.cdc.gov/). Therefore, Cryptococcus infection has attracted increasing attention. *Cryptococcus neoformans* and *Cryptococcus gattii* are the two most common species causing human cryptococcosis^1,2^; Cryptococcus species need to be identified to guide and predict treatment^3^. Gattii and Eeckels reported the first case of *C. gattii* infection in 1970. Since then, there have only been a few reports of its occurrence. *C. gattii* was initially called *C. neoformans* var. *gattii* because its characteristics were very similar to those of *C. neoformans*. In 2002, Kwon-Chung et al.^4^ found that there were some differences in phenotype, biology, and genetic taxonomy between *C. neoformans* and *C. neoformans* var. *gattii* ; thus, *C. gattii* was reclassified as a single species.

Traditional detection methods for cryptococcus, such as ink staining and antigen kit testing, are relatively fast but cannot distinguish between *C. neoformans* and *C. gattii*^2,5^. Polymerase chain reaction (PCR), matrix-assisted laser desorption/ionization time-of-flight mass spectrometry (MALDI TOF MS), gene chips, and second-generation sequencing technology have all been used for clinical identification of Cryptococcus and can distinguish between *C. neoformans* and *C. gattii*; however, the equipment costs are very high^6–10^. The surface-enhanced Raman scattering (SERS) effect refers to the Raman scattering signal of adsorbed molecules due to the enhancement of an electromagnetic field on or near the sample surface, or in the excitation region of specific metal conductors or sol that have been specially prepared^11^. SERS offers several advantages for fungal identification that can make up for the deficiencies of the above detection methods. For example, SERS spectra offer spectral fingerprints unique to the constituent molecules. SERS offers qualitative and quantitative detection of the analyte because it has high specificity^12^. The SERS spectral bands are narrow with high resolution of spectral peaks, thus minimizing fluorescence background interference^13^. The characteristic spectra facilitate simultaneous detection of multiple components. Surface-enhanced Raman spectroscopy offers biological testing and is relatively inexpensive and portable^14^.

It is surprising that there are no reports using SERS for *C. neoformans* and *C. gattii* detection. Although SERS can identify pathogens, further development and improvement is needed before clinical application. This is particularly true for cryptococcus—a special fungal pathogen that contains a capsule^15,16^.

Silver nanoparticles (AgNPs) are widely used as standard SERS substrates^17,18^. The most common approach uses 1% sodium citrate mixed with 1.69 mM AgNO_3_ with heating at 100 °C^19^. The enhancement and reproducibility with anionic analytes are low due to electrostatic repulsion from the sodium citrate on the AgNPs^20,21^. We first measured the zeta potential of *C. neoformans* and *C. gattii* and found that both had negative charges on their surfaces. We previously prepared positively charged silver nanoparticles (AgNPs^+^) with a NaBH_4_ reducing agent; the AgNPs^+^ could adsorb onto Cryptococcus via electrostatic interactions but the stability of this substrate was relatively poor^22^. To improve the stability of the substrate, AgNPs^+^ were synthesized with cetyl trimethylammonium bromide (CTAB) as a cationic surfactant^23^. Herein, we report a rapid detection method for Cryptococcus by SERS using positively charged silver nanoparticles for the first time. After obtaining Cryptococcus spectral data, we used orthogonal partial least-squares discriminant analysis (OPLS-DA), an orthogonal, partial further-squares discriminant analysis algorithm, to analyze the spectral data.

## Results

### Structural characterization of nanomaterials

**Figure 1:**
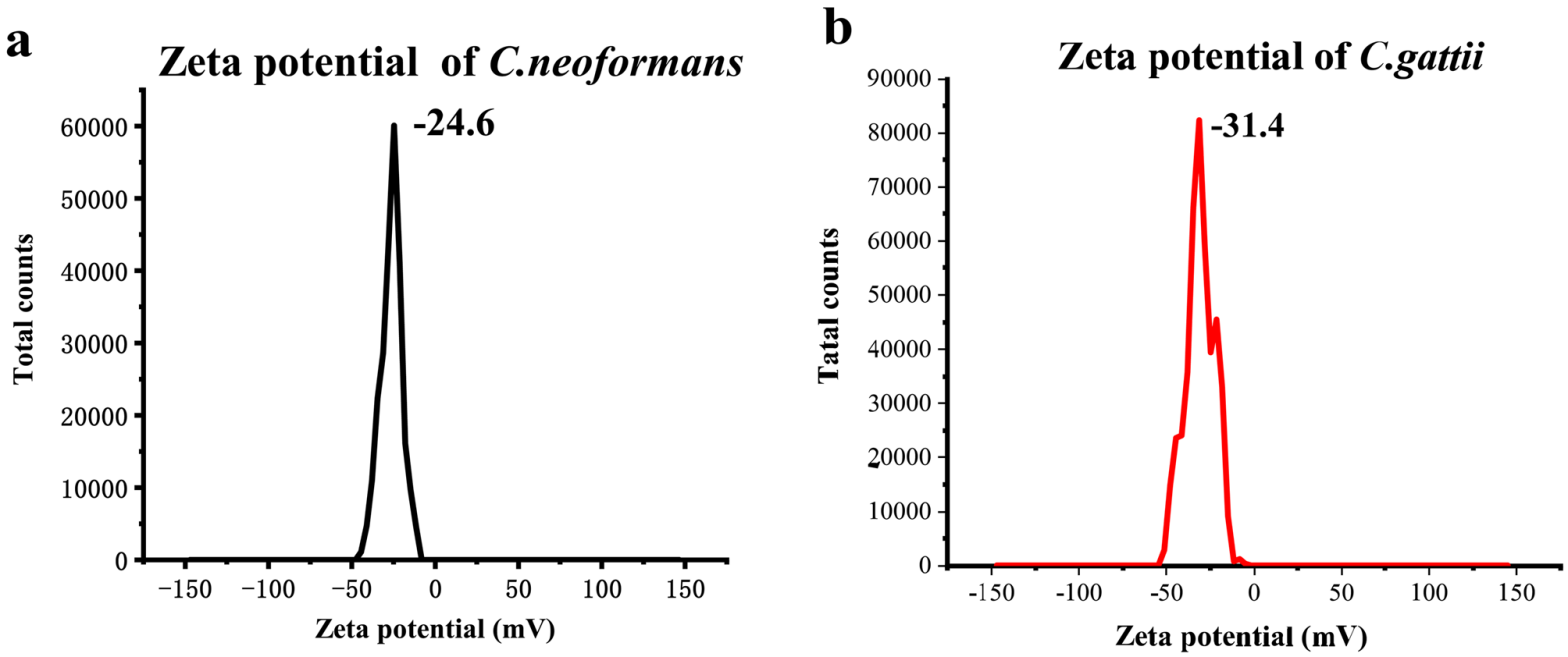
Zeta potential of *C. neoformans* and *C. gattii*. (**a**) Zeta potential of *C. neoformans*, where its surface carries a negative charge. (**b**) Zeta potential of *C. gattii*, where its surface carries a negative charge.

**Figure 2:**
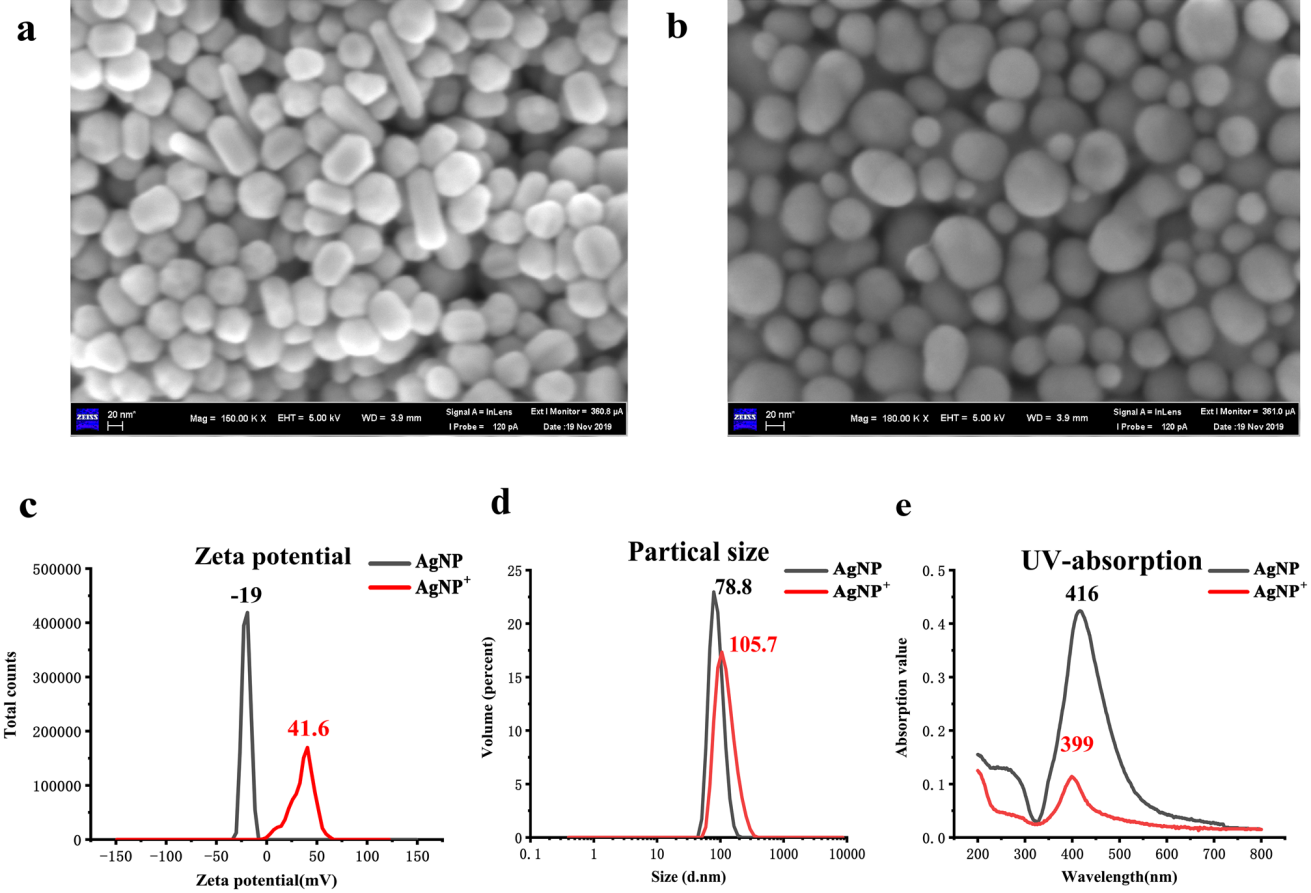
Structural characterization of the nanomaterials. (**a**) Scanning electron microscopy image of the AgNPs. The size and distribution of the AgNPs are homogeneous, and rods of nanoscale silver were produced. (**b**) Scanning electron microscopy image of the spherical AgNPs^+^. (**c**)The AgNPs zeta potential is negative and the AgNPs^+^ zeta potential is positive. (**d**) The AgNPs particle size is 78.8 nm and the AgNPs^+^ particle size is 105.7 nm. (**e**) The UV-absorption spectra of the AgNPs and AgNPs + , where the ultraviolet absorption peaks are both near 400 nm.

**Figure 3:**
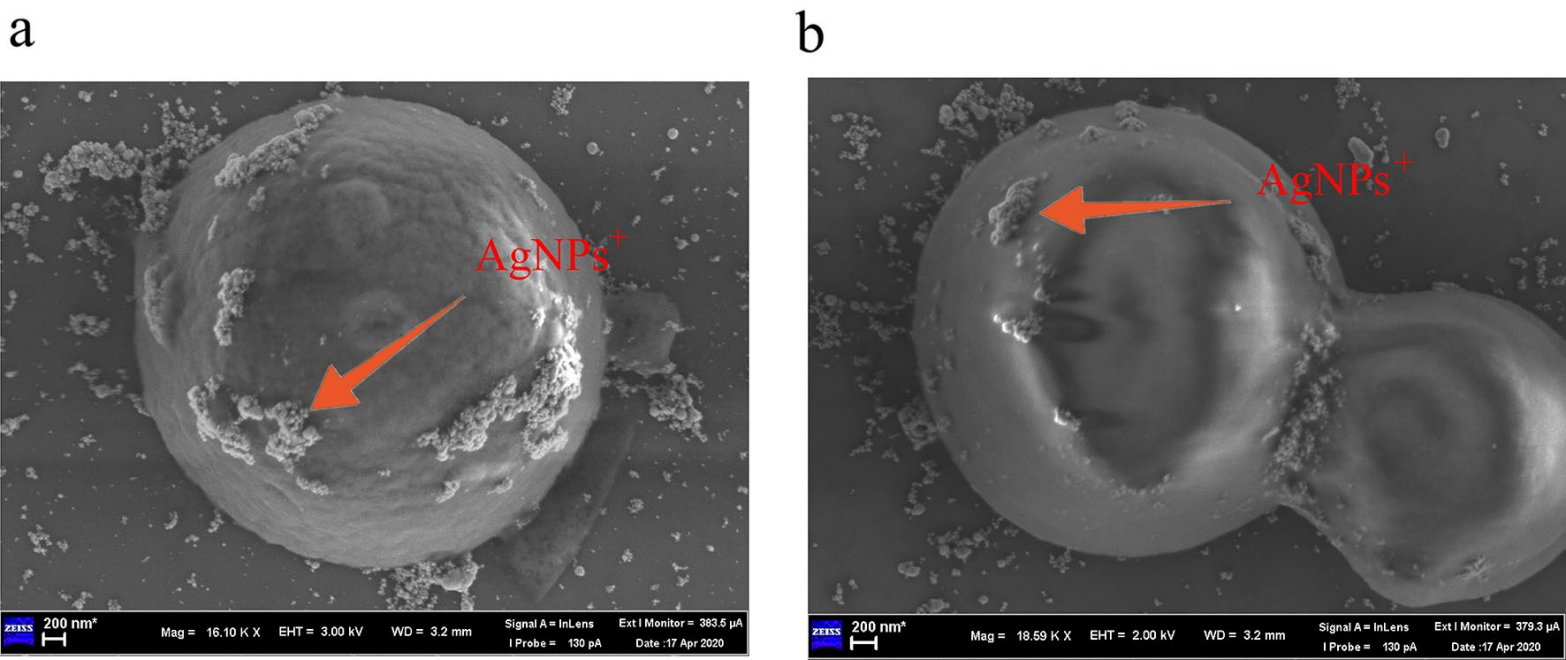
Scanning electron microscopy image of the AgNPs^+^ adsorbed on the surface of *C. neoformans* and *C. gattii*. (**a**) The AgNPs^+^ adsorbed on the surface of *C. neoformans.* (**b**) The AgNPs^+^ adsorbed on the surface of *C. gattii.*

Figure 1a,b show that *C. neoformans* and *C. gattii* are negatively charged. Figure 2a shows a SEM image of the AgNPs. The size and distribution of the AgNPs are homogeneous, and rods of nanoscale silver were produced. Figure 2b shows a SEM image of the AgNPs^+^ and that the AgNPs^+^ are spherical. Figure 2c shows that the AgNPs zeta potential is negative and the AgNPs^+^ zeta potential is positive. The AgNPs particle size is 78.8 nm and the AgNPs^+^ particle size is 105.7 nm, as shown in Fig. 2d. The UV-absorption spectra of the AgNPs and AgNPs^+^ are shown in Fig. 2e, and the ultraviolet absorption peaks are both near 400 nm. Figure 3 shows that the AgNPs^+^ were adsorbed onto the surface of *C. neoformans* and *C. gattii*.

### Raman enhancement effect

**Figure 4:**
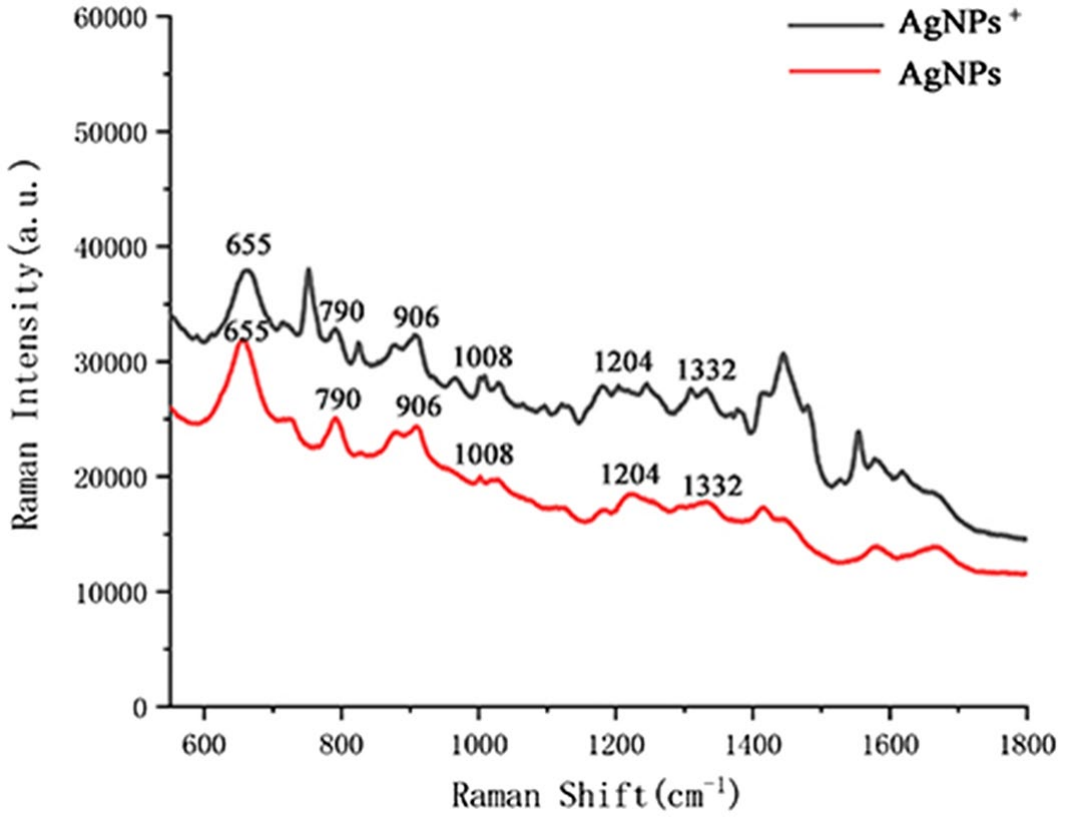
Surface-enhanced Raman scattering spectra measured with two different substrates for *C. neoformans* of the same concentration. The positions of each main peak of *C. neoformans* and *C. gattii* were similar, but the Raman shifts at 655, 790, 906, 1,008, 1,204 and 1,332 cm^−1^ were significantly enhanced.

*Cryptococcus neoformans* was used to verify the Raman enhancement effect of the AgNPs^+^. The same volume of AgNPs and AgNPs^+^ were mixed with the same volume of fungal liquid at a 1:1 ratio, and their Raman signal intensities were compared under the same excitation power and integration time. Figure 4 shows that the Raman signal of each peak was stronger with the AgNPs^+^. Therefore, the AgNPs^+^ was used as the enhancement substrate to distinguish the two species of Cryptococcus.

### SERS technology and multivariate statistical analysis

**Figure 5:**
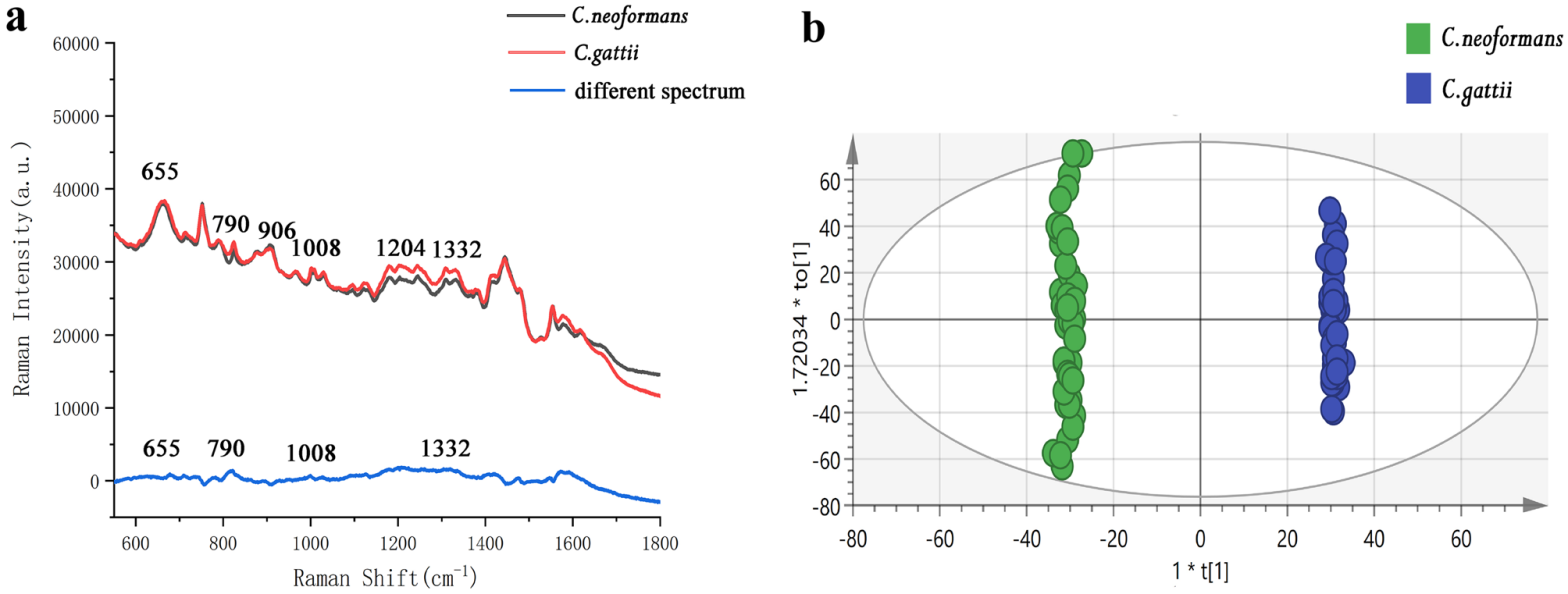
Distinguishing *C. neoformans* and *C. gattii.* (**a**) Surface-enhanced Raman scattering spectrum of *C. neoformans* and *C. gattii* and difference spectrum. Their peaks have differences at 655 cm^−1^, 790 cm^−1^, 1,008 cm^−1^, and 1,332 cm^−1^. The peak bands at 655 cm^−1^ are assigned to Guanine and tyrosine, the peak located at 790 cm^−1^ is assigned to Cytosine and uracil, the peak located at 1,008 cm^−1^ is assigned to Phenylalanine, galactomannan and C–C aromatic ring stretching, and the peak located at 1,332 cm^−1^ is assigned to Adenine, guanine and CH deformation. (**b**) Orthogonal partial least-squares discriminant analysis of *C. neoformans* and *C. gattii* via SERS spectra, where each point represents a sample. The data points are well separated on both sides of the central axis. There is no intersection and they are concentrated on both sides.

The Raman spectra were averaged from the Raman spectra of each Cryptococcus species; the x coordinate is the Raman shift, and the y coordinate is the Raman intensity. Figure 5a compares the average SERS spectra of *C. neoformans* and *C. gattii*. The red line is the mean value of the SERS spectrum of *C. neoformans*, the black line is from the SERS spectrum of *C. gattii*, and the blue line is the difference between them. As shown in Fig. 4, the positions of each main peak of *C. neoformans* and *C. gattii* were similar, and the Raman shifts at 655, 790, 906, 1,008, 1,204 and 1,332 cm^−1^ were significantly enhanced. The physiological structure and biochemical properties of *C. neoformans* and *C. gattii* are very similar. This explains why *C. gattii* remained a variant of *C. neoformans* until 2002^4^. However, their peaks have differences at 655 cm^−1^, 790 cm^−1^, 1,008 cm^−1^, and 1,332 cm^−1^. The peak bands at 655 cm^−1^ are assigned to Guanine and tyrosine, the peak located at 790 cm^−1^ is assigned to Cytosine and uracil, the peak located at 1,008 cm^−1^ is assigned to Phenylalanine, galactomannan and C–C aromatic ring stretching, and the peak located at 1,332 cm^−1^ is assigned to Adenine, guanine and CH deformation.

OPLS-DA was used to further distinguish *C. neoformans* and *C. gattii*, as the fingerprints are different between the two species of cryptococcus. OPLS-DA based on the SIMCA14.1 software was performed on the Raman spectrum data from all measurements. Figure 5b shows an ellipse at the 95% confidence interval of the sample analysis results, where each point represents a sample^24^. The data points are well separated on both sides of the central axis. There is no intersection and they are concentrated on both sides. The OPLS-DA model 1 (Table 1) showed that R^2^X (cum) = 0.986, R^2^Y (cum) = 0.999, and Q^2^ (cum) = 0.998, indicating that the quality parameters of the model were good. Here, R^2^X represents the interpretation rate of the model in the X-axis direction, i.e., 98.6%. Term R^2^Y describes the model in the Y-axis direction, i.e., 99.9%, and Q^2^ is the prediction rate of the model, i.e., 99.8%^25^.

**Table 1:** Parameters of OPLS-DA. Model 1: distinguishing *C. neoformans* and *C. gattii*. Models 2–11: tenfold crossover validation.

Model	Type	Number	R^2^X (cum)	R^2^Y (cum)	Q^2^ (cum)
1	OPLS-DA	80	0.986	0.999	0.998
2	OPLS-DA	72	0.501	0.937	0.933
3	OPLS-DA	72	0.985	0.998	0.998
4	OPLS-DA	72	0.986	0.998	0.998
5	OPLS-DA	72	0.986	0.999	0.998
6	OPLS-DA	72	0.506	0.948	0.947
7	OPLS-DA	72	0.505	0.943	0.932
8	OPLS-DA	72	0.498	0.922	0.916
9	OPLS-DA	72	0.503	0.92	0.918
10	OPLS-DA	72	0.501	0.919	0.917
11	OPLS-DA	72	0.502	0.918	0.916

The classification ability of the model was evaluated using a tenfold crossover validation with SIMCA14.1. All the SERS spectrum data of *C. neoformans* and *C. gattii* were divided into 10 parts. Nine parts were successively used as training data, and one part served as the test data. The average accuracy of the training and test data were obtained after 10 operations. Tables 1 and 2 (Model 2–11) show that the average accuracy of the training and test data are 100% and 100%, respectively, which indicates that the model has good classification ability^26,27^.

**Table 2:** Results of the tenfold crossover validation of *C. neoformans* and *C. gattii* based on orthogonal partial least-squares discriminant analysis.

Time	Accuracy of training data (%)	Accuracy of test data (%)
1	100	100
2	100	100
3	100	100
4	100	100
5	100	100
6	100	100
7	100	100
8	100	100
9	100	100
10	100	100
Mean	100	100

## Discussion

Cryptococcosis is a global, invasive fungal infectious disease caused by Cryptococcus. Treatment of this disease is still extremely challenging. *C. neoformans* infection accounts for the majority of cryptococcosis, whereas *C. gattii* infection is relatively rare^28^. *C. neoformans* causes infections mainly in the central nervous system; pulmonary infection accounts for only 35% of all infected patients. In an in vivo microscopy experiment conducted by Ngamskulrungroj et al.^29,30,31^, there was no significant difference in the clinical symptoms between patients with these two cryptococcal infections, and a differential diagnosis based on clinical symptoms was difficult^32,33,34^. However, *C. gattii* requires longer periods of antifungal therapy, which motivates our efforts for discriminating between the two species.

Traditional methods include ink staining and antigen kit testing^5^. Although the ink detection method of cerebrospinal fluid (CSF) is simple and fast, distinguishing between *C. neoformans* and *C. gattii* is difficult^2^. Cryptococcal antigen kits can use latex agglutination and enzyme-linked immunosorbent assays to study the cryptococcal polysaccharide capsular antigen (CrAg) composition in blood. This method offers rapid diagnosis within 2–3 h, and it is more sensitive than direct staining microscopy. However, most of the antiserum used is based on the *C. neoformans* antigen, and it is possible to miss the detection of *C. gattii*^35^. Antigen clearance kinetics are slower than treatment response, and continuous monitoring has limited value. Regarding molecular biology methods, PCR gene sequences using specific gene segments can be used for identification and typing. These are sensitive and specific and can be used to genotype *C. neoformans* and *C. gattii*^36^. However, PCR is time-consuming and prone to contamination^37^. MALDI-TOF MS can also classify cryptococci and can reliably display the genetic and evolutionary relationships between species and within species. MALDI-TOF MS is more accurate and faster than gene sequencing^38^. However, it is expensive and only research hospitals can support a mass spectrometer. Gene chips are also expensive and difficult to use in routine clinical care, and second-generation sequencing requires bioinformatics professionals.

All current cryptococcus detection methods have limitations, and this study used surface enhanced Raman spectroscopy to detect cryptococcus. Versus other detection methods for *C. neoformans* and *C. gattii*^5–10^*,* SERS exhibits high specificity with low costs and fast analysis times. SERS can quickly identify and distinguish *C. neoformans* and *C. gattii*. Here, AgNPs^+^ were formed from AgNO_3_ with CTAB and NaBH_4_. We found that the AgNO_3_ concentration could be used to tune the size of the AgNPs^+^^35^. This method is simple and easy to repeat. Its repeatability compensates for the difficult nanomaterial synthesis. The preparation is convenient and can be easily promoted and applied in a clinical setting. The SEM image in Fig. 2b shows the characteristic structure of the AgNPs^+^. The AgNPs^+^ were spherical, colloidally stable, and approximately 110 nm in diameter. The AgNPs^+^ served as the SERS substrate. We measured the spectra of high-quality *C. neoformans* and *C. gattii*, and Fig. 5a compares the average SERS spectra of *C. neoformans* and *C. gattii*.

The spectra for the two cryptococcus show few differences because they are closely related. Therefore, it is necessary to use an OPLS-DA analysis tool. Figure 5b shows the OPLS-DA score diagram of the SERS spectra of *C. neoformans* and *C. gattii*. All the data points were distributed on both sides of the central axis with no intersection, indicating that OPLS-DA could distinguish the SERS spectra of *C. neoformans* and *C. gattii* well. In addition, as shown in Table 1, R2X(cum) = 0.986, R2Y(cum) = 0.999 and Q2(cum) = 0.998, indicating that the quality parameters of the established model are good. Table 2 shows that the average accuracy of training and test data is 100% and 100%, respectively, illustrating that the model has good classification ability.

We have initiated the application of SERS for cryptococcus detection. SERS can be used for rapid and nondestructive detection of Cryptococcus with relatively low equipment cost. Due to the special properties of the capsule, cryptococcus is difficult to detect relative to other fungi such as candida and mold. Therefore, many detection methods that can be applied to detect common fungi cannot be used to detect cryptococcus. This highlights the universal applicability of Raman spectroscopy to detect pathogenic fungi. The OPLS-DA data shows that Raman can clearly distinguish between two cryptococcus species with very similar properties, which is an excellent result.

## Conclusions

We report a novel detection method to identify and discriminate *C. neoformans* and *C. gattii*. The positively charged nanoparticles are shown to increase the intensity of the Raman spectra acquired from Cryptococcus. A classification model to discriminate the SERS spectra of *C. neoformans* and *C. gattii* was then built using OPLS-DA. This model is capable of explaining over 99% of the variance in the data and achieves a 99.8% predictive rate of discrimination. These results indicate that a SERS-based diagnostic tool may be a powerful technique for diagnosing cryptococcosis.

## Methods

### Ethical statement

This study has been approved by the ethics review committee of Shanghai hospital, China. Comply with the World Medical Association Declaration of Helsinki, the relevant Chinese laws and regulations, and other ethical principles to protect the rights and interests of the subjects, as well as the regulations of the ethics committee of Shanghai (accession number: 2013SMMU-LL013). Signed informed consent were acquired from all patients with the admission number: 277311, 296013, 454964 and 484728.

### Chemicals

AgNO_3_ (99%) was purchased from Beijing Modern Oriental Trading Co., Ltd., NaBH_4_ (99%), CTAB was from Sigma-Aldrich Trading Co., Ltd. (Shanghai, China), and ammonium hydroxide (NH_4_OH) was from Xilong Chemical Group Co., Ltd. (China). All commercial chemical reagents were used directly without further purification. The monocrystalline Si used to detect the Raman signal was supplied by Zhejiang Lijing Silicon Materials Co., Ltd. (China). In addition, the Sabouraud Dextrose agar (SDA) medium used to culture Cryptococcus was purchased from Shanghai Comaga Microbial Technology Co., Ltd. (China). The Milli-Q water was obtained from Millipore Systems Corp. (USA).

### Instrumentation

The ultraviolet–visible-light (UV–Vis) spectrophotometer was a Shimadzu 2600 (Shimadzu, Japan). Morphology studies used a Hitachi h-7650 (Hitachi, Japan) scanning electron microscope (SEM), a Zeiss thermal field emission scanning electron microscope-Merlin (Carl Zeiss, Germany), and a JEOL 2010 high-resolution transmission electron microscope (HR-TEM; Nippon Electronics Co., Ltd., Japan). The zeta potentials were measured with a Zetasizer Nano ZSP analyzer (Malvern, UK). SERS measurements used the i-raman Plus bws465-785 h Raman spectrometer (B&W Tek, USA). Other instruments included a MiniSpin centrifuge (Eppendorf, Germany) and a zncl-bs230 *230 heated electromagnetic stirrer (Beijing Century Huake Co., China).

### Synthesis of the substrate

AgNPs^+^ colloids were prepared according to previous methods by van Lierop et al.^17^ and Chen et al.^18^. The AgNPs^+^ diameters could be increased to more than 100 nm^17,18^. Solution A was prepared first, as follows: 3.26 mM AgNO_3_, 0.4 M NH_4_OH, and 0.5 mM CTAB (cetyl trimethyl ammonium bromide) were added to 20 mL of ultrapure water. Solution B was then prepared as follows: 8 mM NaBH_4_ and 0.5 mM CTAB were added to 20 mL of ultrapure water. Solutions A and B were placed in an ice bath for 15 min to slow the reaction. Solution A was then poured into a 50-mL conical flask that was vigorously stirred on a heated electromagnetic stirrer at 700 rpm. Solution B was then poured into solution A dropwise and stirred on a heated electromagnetic mixer for 4.5 h; this process was repeated in an ice bath. The conical flask was sealed during the stirring process to prevent the silver from being oxidized. The stirring was stopped after 4.5 h, and the mixture was heated at 100℃ for 12 min to remove the remaining NH_3_ and NaBH_4_. The heat was then turned off, and the stirring was continued until the mixture cooled to room temperature. Ultrapure water was then added to a total volume of 40 mL; 1.5 mL of the solution was placed in a 1.5 mL polystyrene tube and centrifuged at 7,500 rpm for 7 min. After centrifugation, 1.4 mL of the supernatant was discarded, and the remaining solution was mixed. The resulting AgNPs^+^ were stored in a refrigerator at 4 °C.

### Strain collection and identification

Clinical isolates of *C. neoformans* and *C. gattii* were collected as cerebrospinal fluid (CSF) at Shanghai Changzheng Hospital, China. The specimens were from various media including blood plate medium, chocolate agar plate medium, sabouraud dextrose agar (SDA) medium, or isolated from a single colony. Selected fungal colonies were cultured for 48 h in ambient air at 25 °C in SDA medium. The fungal suspension was identified using India ink staining, a lateral flow assay (LFA) for cryptococcal antigen (CrAg) detection (ImmunoMycologics, Inc, Norman, OK, USA) and using an ABI_3730xl sequenator (Genomic Instrument, USA) to specifically sequence the intergenic spacer 1 (IGS1) region of the nuclear ribosomal rRNA gene of the fungal culture.

### Preparation of SERS detection samples

**Figure 6:**
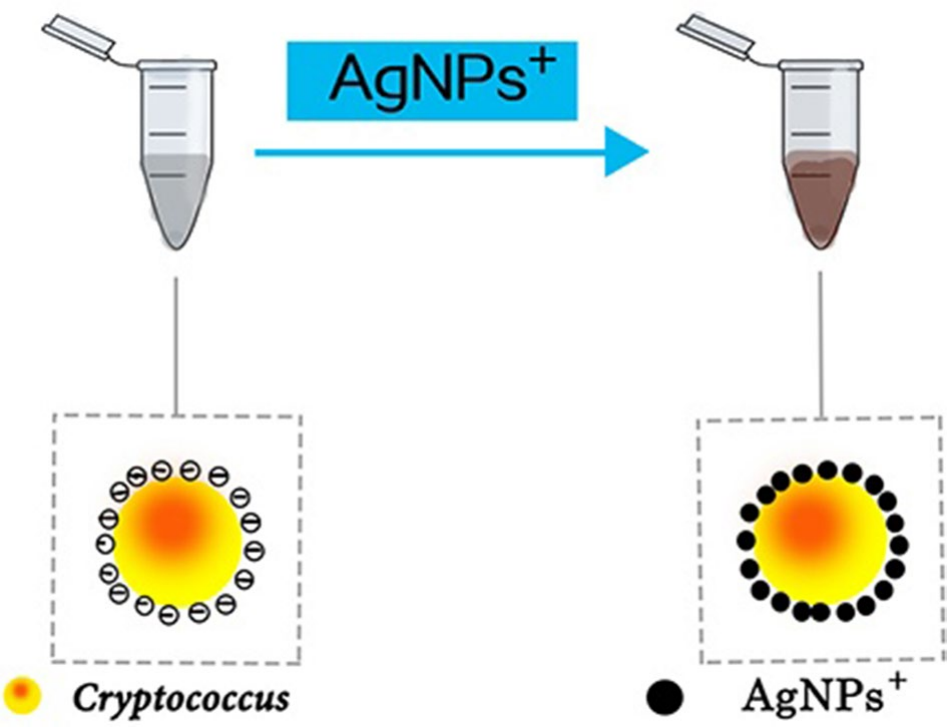
Positively charged AgNPs^+^ mixed with negatively charged *C. neoformans.*

*Cryptococcus gattii* is very clinically rare. Two strains of *C. neoformans* and two strains of *C. gattii* were selected and grown in SDA medium for 48 h at 25 °C. Approximately 50 μL of Cryptococcus colonies were mixed with 100 μL of ultrapure water and oscillated for 2 min to ensure complete mixing. The number of *C. neoformans* and *C. gattii* in the solution was calculated by a fungal counter as 1.03 × 10^9^ and 1.05 × 10^9^ cells mL^–1^, respectively, followed by mixing of 10 μL of fungus liquid with 10 μL of the pre-prepared AgNPs^+^ as shown in Fig. 6. Next, 2 μL of the mixture was placed on the monocrystalline silicon. They were detected by Raman spectroscopy (i-raman Plus BWS465785H) after the mixture completely dried.

### SERS measurements

The Raman spectrometer used in this experiment has a maximum laser power of 275 mW at 785 nm with a light spot diameter of 105 μm. The excitation power was 20% of the maximum power, and the integration time was 15 s. We used the white light function to find the darkest area on the wafer, and 20 replicate SERS spectra were recorded. The processed spectra were used to compare the average Raman data from *C. neoformans* and *C. gattii*. The Cryptococcus mixtures were placed on a silicon chip and studied with a microscope; 20 different fields-of-view were studied. The total acquisition time of the SERS spectrum was 45 min, including the time of centrifugation of the substrate, sample preparation, and spectrum testing.

### Statistical analysis

The OPLS-DA spectral data were collected with SIMCA 14.1 (Umetrics, Umea, Sweden) software. OPLS-DA is a supervised discriminant analysis method that predicts differences in fungi species by establishing the relationship model between the expression level of metabolites and the fungi species. The OPLS-DA model is established for comparisons between two groups, and the parameter evaluation of the model is presented in the form of a table in which R^2^X and R^2^Y represent the interpretation rate of the established model to the X and Y matrix, respectively; term Q^2^ indicates the prediction ability of the model^24^. The fitting accuracy of the model improves as R^2^ and Q^2^ approach 1. Lower values of R^2^ and Q^2^ lead to worse model fitting accuracy. A fitting accuracy of R^2^ and Q^2^ above 0.5 (50%) is good, and higher than 0.4 is acceptable.

The accuracy of the OPLS-DA model for classification of unknown samples was predicted via a tenfold crossover validation. The classification ability of the model was also evaluated by a tenfold crossover examination. The SERS data of all *C. neoformans* and *C. gattii* were divided into 10 parts; nine parts were successively used as training data and one part served as the test data. After 10 operations, the average accuracy of the training and test data was obtained. The mean value of the accuracy (or error rate) of the results of the tenfold crossover validation was used to estimate the accuracy of the algorithm.

## References

[1] May RC, Stone NRH, Wiesner DL, Bicanic T, Nielsen K (2016). Cryptococcus: From environmental saprophyte to global pathogen. Nat. Rev. Microbiol..

[2] Maziarz EK, Perfect JR (2016). Cryptococcosis. Infect. Dis. Clin. N. Am..

[3] Chen M (2016). Cryptococcosis and tuberculosis co-infection in mainland China. Emerg. Microbes Infect..

[4] Kwon-Chung KJ, Boekhout T, Fell JW, Diaz M (2002). Proposal to conserve the name *Cryptococcus gattii* against *C. hondurianus* and *C. bacillisporus* (*Basidiomycota*, *Hymenomycetes*, *Tremellomycetidae*). Taxon.

[5] Kammalac Ngouana T (2015). Cryptoccocal meningitis in Yaoundé (Cameroon) HIV infected patients: Diagnosis, frequency and *Cryptococcus neoformans* isolates susceptibility study to fluconazole. J. Mycol. Med..

[6] Morrell M, Fraser VJ, Kollef MH (2005). Delaying the empiric treatment of candida bloodstream infection until positive blood culture results are obtained: A potential risk factor for hospital mortality. Antimicrob. Agents Chemother..

[7] Bian F (2015). Study on genotype and virulence of *Cryptococcus neoformans* and *Cryptococcus gattii* clinical isolates in Guigang, Guangxi Zhuang Autonomous Region. Zhonghua Liu Xing Bing Xue Za Zhi..

[8] Angeletti S (2017). Matrix assisted laser desorption time of flight mass spectrometry (MALDI-TOF MS) in clinical microbiology. J. Microbiol. Methods.

[9] Jia W (2017). Application of gene chip technology for acupuncture research over the past 15 years. Zhongguo Zhen Jiu..

[10] Clark AE, Kaleta EJ, Arora A, Wolk DM (2013). Matrix-assisted laser desorption ionization-time of flight mass spectrometry: A fundamental shift in the routine practice of clinical microbiology. Clin. Microbiol. Rev..

[11] Cottat M (2015). High sensitivity, high selectivity SERS detection of MnSOD using optical nanoantennas functionalized with aptamers. J. Phys. Chem. C.

[12] Driskell JD, Kwarta KM, Lipert RJ, Porter MD, Neill JD, Ridpath JF (2005). Low-level detection of viral pathogens by a surface-enhanced Raman scattering based immunoassay. Anal. Chem..

[13] Gherman AMR, Dina NE, Chiș V, Wieser A, Haisch C (2019). Yeast cell wall–silver nanoparticles interaction: A synergistic approach between surface-enhanced Raman scattering and computational spectroscopy tools. Spectrochim. Acta A Mol. Biomol. Spectrosc..

[14] Mosier-Boss PA (2017). Review on SERS of bacteria. Biosensors (Basel).

[15] Tu Q, Chang C (2012). Diagnostic applications of Raman spectroscopy. Nanomed. Nanotechnol. Biol. Med..

[16] Efrima S, Zeiri L (2009). Understanding SERS of bacteria. J. Raman Spectrosc..

[17] van Lierop D (2012). Positively charged silver nanoparticles and their effect on surface-enhanced Raman scattering of dye-labelled oligonucleotides. Chem. Commun..

[18] Chen X (2019). Surface-enhanced Raman scattering method for the identification of methicillin-resistant *Staphylococcus aureus* using positively charged silver nanoparticles. Microchim. Acta.

[19] Kahraman M, Zamaleeva AI, Fakhrullin RF, Culha M (2009). Layer-by-layer coating of bacteria with noble metal nanoparticles for surface-enhanced Raman scattering. Anal. Bioanal. Chem..

[20] Félix-Rivera H, González R, Rodríguez GDM, Primera-Pedrozo OM, Ríos-Velázquez C, Hernández-Rivera SP (2011). Improving SERS detection of *Bacillus thuringiensis* using silver nanoparticles reduced with hydroxylamine and with citrate capped borohydride. Int. J. Spectrosc..

[21] Preciado-Flores S (2011). SERS spectroscopy and SERS imaging of *Shewanella oneidensis* using silver nanoparticles and nanowires. Chem. Commun..

[22] Tan S, Erol M, Attygalle A, Du H, Sukhishvili S (2007). Synthesis of positively charged silver nanoparticles via photoreduction of AgNO_3_ in branched polyethyleneimine/HEPES solutions. Langmuir.

[23] Sui ZM (2006). Capping effect of CTAB on positively charged Ag nanoparticles. Phys. E Low Dimens. Syst. Nanostruct..

[24] Gillibert R, Triba MN, Lamy de la Chapelle M (2018). Surface enhanced Raman scattering sensor for highly sensitive and selective detection of ochratoxin A. Analyst.

[25] Jolayemi OS, Ajatta MA, Adegeye AA (2018). Geographical discrimination of palm oils (*Elaeis guineensis*) using quality characteristics and UV–visible spectroscopy. Food. Sci. Nutr..

[26] Abbas MM, Mohie-Eldin MM, El-Manzalawy Y (2015). Assessing the effects of data selection and representation on the development of reliable *E. coli* sigma 70 promoter region predictors. PLoS ONE.

[27] Simon RM, Subramanian J, Li MC, Menezes S (2011). Using cross-validation to evaluate predictive accuracy of survival risk classifiers based on high-dimensional data. Brief Bioinform..

[28] Del Valle L, Piña-Oviedo S (2006). HIV disorders of the brain; pathology and pathogenesis. Front. Biosci..

[29] Ngamskulrungroj P, Chang Y, Sionov E, Kwon-Chung KJ (2012). The primary target organ of *Cryptococcus gattii* is different from that of *Cryptococcus neoformans* in a murine model. mBio.

[30] Charlier C, Chrétien F, Baudrimont M, Mordelet E, Lortholary O, Dromer F (2005). Capsule structure changes associated with *Cryptococcus neoformans* crossing of the blood–brain barrier. Am. J. Pathol..

[31] Nassif X, Bourdoulous S, Eugène E, Couraud P-O (2002). How do extracellular pathogens cross the blood–brain barrier?. Trends Microbiol..

[32] World Health Organization. *Guidelines for the DIAGNOSIS, prevention, and Management of Cryptococcal Disease in HIV-Infected Adults, Adolescents and Children, March 2018: Supplement to the 2016 Consolidated Guidelines of the Use of Antiretroviral Drugs for TREATING and Preventing HIV Infection* (World Health Organization, Geneva, 2018). 30285342

[33] Darras-Joly C (1996). *Cryptococcus neoformans* infection in France: Epidemiologic features of and early prognostic parameters for 76 patients who were infected with human immunodeficiency virus. Clin. Infect. Dis..

[34] Tay ST, Rohani MY, Soo Hoo TS, Hamimah H (2009). Epidemiology of cryptococcosis in Malaysia. Mycoses.

[35] Lindsley MD (2011). Evaluation of a newly developed lateral flow immunoassay for the diagnosis of cryptococcosis. Clin. Infect. Dis..

[36] Rivera V, Gaviria M, Muñoz-Cadavid C, Cano L, Naranjo T (2015). Validation and clinical application of a molecular method for the identification of *Cryptococcus neoformans*/*Cryptococcus gattii* complex DNA in human clinical specimens. Braz. J. Infect. Dis..

[37] Boondireke S (2010). Evaluation of sensitivity of multiplex PCR for detection of *Mycobacterium tuberculosis* and *Pneumocystis jirovecii* in clinical samples. J. Clin. Microbiol..

[38] Panda A (2015). MALDI-TOF mass spectrometry for rapid identification of clinical fungal isolates based on ribosomal protein biomarkers. J. Microbiol. Methods.

[39] Wang J (2015). Magnetically assisted surface-enhanced raman spectroscopy for the detection of *Staphylococcus aureus* based on aptamer recognition. ACS Appl. Mater. Interfaces.

